# Mn-Doped Carbon Dots as Contrast Agents for Magnetic Resonance and Fluorescence Imaging

**DOI:** 10.3390/ijms26136293

**Published:** 2025-06-29

**Authors:** Corneliu S. Stan, Adina Coroaba, Natalia Simionescu, Cristina M. Uritu, Dana Bejan, Laura E. Ursu, Andrei-Ioan Dascalu, Florica Doroftei, Marius Dobromir, Cristina Albu, Conchi O. Ania

**Affiliations:** 1 Centre of Advanced Research in Bionanoconjugates and Biopolymers, “Petru Poni” Institute of Macromolecular Chemistry, 41A Grigore Ghica Voda Alley, 700487 Iasi, Romania; adina.coroaba@icmpp.ro (A.C.); natalia.simionescu@icmpp.ro (N.S.); cristina-mariana.uritu@umfiasi.ro (C.M.U.); bejan.dana@icmpp.ro (D.B.); ursu.laura@icmpp.ro (L.E.U.); andrei.dascalu@icmpp.ro (A.-I.D.); florica.doroftei@icmpp.ro (F.D.); albuc@ymail.com (C.A.); 2 Faculty of Chemical Engineering and Environmental Protection, “Gheorghe Asachi” Technical University of Iasi, 73 D. Mangeron Ave., 700050 Iasi, Romania; 3 Advanced Center for Research and Development in Experimental Medicine “Prof. Ostin C. Mungiu” (CEMEX), “Grigore T. Popa” University of Medicine and Pharmacy, 16 Universitatii Street, 700115 Iasi, Romania; 4 Science Research Department, Institute of Interdisciplinary Research, Research Center on Advanced Materials and Technologies (RAMTECH), “Alexandru I. Cuza” University of Iasi, 11 Carol I Boulevard, 700506 Iasi, Romania; marius.dobromir@uaic.ro; 5 CEMHTI (CNRS, UPR 3079), Université d’Orléans, 45100 Orleans, France

**Keywords:** carbon nanodots, manganese complexes, diphenylhydantoin, magnetic resonance imaging, contrast agents, fluorescence imaging

## Abstract

Carbon nanodots have recently attracted attention as fluorescence imaging probes and magnetic resonance imaging (MRI) contrast agents in diagnostic and therapeutic applications due to their unique optical properties. In this work we report the synthesis of biocompatible Mn (II)-doped carbon nanodots and their performance as fluorescence and MRI contrast agents in in vitro assays. The thermal decomposition of a Diphenylhydantoin–Mn(II) complex assured the incorporation of manganese (II) ions in the carbon dots. The obtained materials display a favorable spin density for MRI applications. The synthesized Mn(II)-CNDs also displayed remarkable photoluminescence, with a bright blue emission and good response in in vitro fluorescence imaging. Cytotoxicity investigations revealed good cell viability on malignant melanoma cell lines in a large concentration range. A cytotoxic effect was observed for MG-63 osteosarcoma and breast adenocarcinoma cell lines. The in vitro MRI assays demonstrated the potentialities of the Mn(II)-CNDs as T2 contrast agents at low dosages, with relaxivity values higher than those of commercial ones. Due to the simplicity of their synthetic pathway and their low cytotoxicity, the prepared Mn(II)-CNDs are potential alternatives to currently used contrast agents based on gadolinium complexes.

## 1. Introduction

Low-dimensional carbon nanodots (CNDs) are a newer class of carbon materials that consists of a carbon core (amorphous and/or graphitic-like) decorated with avariety of functional groups on the surface [1,2]. They have garnered significant interest in nanomedicine as efficient platforms for both therapeutic and diagnostic applications [3,4,5,6,7]. The most important properties of CNDs in biomedical applications are their tunable photoluminescence upon their composition [8,9], their dual capacity to act as antioxidants and radical scavengers [10,11] and their biocompatibility [6]. In this regard, while most CNDs are generally considered as biocompatible and low/non-toxic, these features strongly depend on the nature of the precursor and the synthetic route [6,12]. The challenge is to control the optical and structural properties of the prepared CNDs. The incorporation of metallic heteroatoms in carbon dots seems a useful strategy to obtain CNDs with modulated optical and structural properties and expand their use in bio-applications [13,14,15]. As a few examples, several studies have reported the synthesis of carbon dots doped with transition metal ions (e.g., Ru-, Fe-, Co-, Ni-) and their application in bimodal medical imaging, biosensors, gene detection, and drug delivery [13,14,15,16,17]. Those studies have pointed out metal-doped carbon dots as competitive materials compared to benchmarks in those fields. The difficulty is to avoid the aggregation of the metallic species in their synthesis, as this is responsible for a heterogeneous surface distribution of metal ions.

Numerous studies also report the anti-tumoral effect of carbon dots and their potential application as fluorescent probes and magnetic resonance imaging (MRI) contrast agents [18,19,20,21,22,23,24]. For instance, magneto-fluorescent Gd-doped CNDs prepared from starch have shown dual modal fluorescence and worked as MRI contrast agents in diagnosis and brain mapping applications [19]. Another recent study shows the potential use of CND micelles prepared from leek seeds in sensor applications and cancer therapy [20]. In a similar approach, CNDs prepared from red beans were studied as anti-tumoral agents on 16 cell lines such as liver/pancreatic cancer cells, intrahepatic cholangiocarcinoma cells, and colorectal adenocarcinoma cells [21]. CNDs prepared through hydrothermal approaches of microcrystalline cellulose were found to have a dual anti-tumoral and microbicide action through the inhibition of the proliferation of hepatocellular carcinoma and several pathogens (e.g., bacteria and fungi) [22]. Cu-doped CNDs have been investigated as inhibitors of breast cancer’s progression [23].

In this context, our recent studies have demonstrated the application of Mn-doped carbon dots obtained from a commercial commodity with a high surface functionalization as theranostic platforms for breast cancer treatment in in vitro/in vivo assays [24]. Based on those previous studies, in this work we explore the synthesis of Mn-doped CNDs from the thermal degradation of a Mn(II) complex and their application as fluorescent probes for fluorescence imaging and as MRI contrast agents. To achieve a homogeneous distribution of the manganese ion, our novel approach is based on the thermal decomposition of the complex Diphenylhydantoin-Mn(II). Diphenylhydantoin (also known as Phenytoin) has a favorable structural configuration able to yield stable transition metal complexes [25,26]. Besides its complexation potential, DPH is biocompatible, since it is used as an anti-seizure and anti-arrhythmias medication. These are important criteria to obtain compatible metal-doped carbon dots for biomedical applications. In this study, the morpho-structural characteristics of the Mn(II)-DPH complex and the Mn-doped CNDs have been investigated in detail, such as their photoluminescence properties, biocompatibility, and cytotoxicity on various cancer cell lines and their efficiency as fluorescent probes and MRI contrast agents from in vitro assays [27,28,29]. Their performance has been compared to that of commercial contrast agents based on gadolinium complexes.

## 2. Results and Discussion

### 2.1. Characterization of the Mn-Doped Carbon Dots

**Figure 1:**
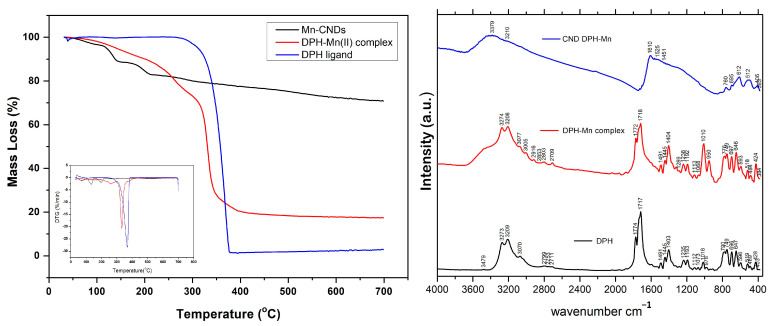
(**left**) Thermogravimetric profiles and DTG plots (inset); (**right**) IR spectra recorded for DPH ligand, Mn(II)-DPH complex, and Mn(II)-CNDs.

The thermogravimetric profiles of the Mn-doped CNDs compared to those of the raw ligand (DPH) and the Mn(II)-DPH complex are shown in Figure 1(left). The degradation profile of DPH shows a sharp decomposition between 300 and 400 °C (maximum at around 375 °C) attributed to its complete degradation (the final mass loss is ca. 97.4 wt.%). When the complex is formed, the profile shows several mass losses and a final mass loss of 82 wt.%; this indicates an increased thermal stability compared to pristine DPH. The first weight loss for the complex Mn(II)-DPH occurs in the 80–240 °C range and accounts for ca. 13.8 wt.%. This is attributed to the loss of water molecules within the outer coordination sphere of the Mn(II) cations and the beginning of the structural decomposition of the material (the expected general formula is Mn(C_15_H_10_N_2_O_2_)_2_(H_2_O)_x_). The second weight loss for the Mn(II)-DPH complex (ca. 14.38 wt.%) is attributed to the continuous intermolecular breaking and incipient decomposition of the molecular structure. The sharp mass loss (50.54 wt.%) at 330 °C accounts for the complete degradation of the complex. It occurs at a lower temperature than DPH, which confirms the binding of the ligand to the manganese ions in the complex. The tail at 375 °C would suggest a minor contribution of free DPH (ca. 5 wt.%).

The thermogravimetric profiles of the Mn(II)-CNDs show several peaks at 150 and 225 °C attributed to the decomposition of labile O- and N- surface moieties. These remnant functional groups are particularly important to achieve photoluminescence properties. The Mn(II)-CNDs showed a final mass loss of ca. 28 wt.%, ca. four times higher than that of the Mn(II)-DPH complex. This indicates a rearrangement of the volatile matter of the complex during the thermal treatment at 490 °C for 10 min. The continuous mass loss above 400 °C indicates the presence of some functional groups and/or volatile matter that would still be decomposed upon further heating over 700 °C.

Figure 1(right) shows the IR spectra of the prepared samples, and Table 1 compiles the main vibration peaks and their attributions to various groups. The spectra of the Mn(II)-DPH complex shows several differences with that of free DPH. For instance, the band located at 3600–3200 cm^−1^ characteristic of OH stretching frequencies indicates the presence of water molecules within the outer coordination sphere of the Mn(II) cations (also observed in the thermogravimetric profiles). The small intensity of the N-H stretch signal (3479 cm^−1^) in DPH ligand was no longer visible in the Mn(II)-DPH complex. Small displacements in the vibration signals of C=N and C=O (e.g., 1770–1600 cm^−1^) observed in the complex are attributed to the presence of the coordinative bonding of nitrogen and oxygen atoms with a central Mn(II) cation. The peak at 1010 cm^−1^ in free DPH decreased significantly for the complex Mn(II)-DPH, while a new band appeared at 950 cm^−1^ in the complex. This can be assigned to (Mn–N) stretching vibrations [30]. Small differences in the fingerprint region 800–400 cm^−1^ are attributed to Mn-N metal–ligand bonds in the complex.

In the Mn(II)-CNDs, a wide hump at 3600–3000 cm^−1^, characteristic of OH stretching in different configurations (OH surface groups) in carbon materials, is observed. The bands at 17,750–1700 cm^−1^ of the C=N and C=O vibrations of the DPH and the complex are no longer observed in the Mn-CDs, but the band at 1610 cm^−1^ characteristic of fused aromatic carbon atoms is pronounced. The bands between 1010 and 900 cm^−1^ assigned to M-N vibrations are also not visible after the thermal treatment of the complex.

**Figure 2:**
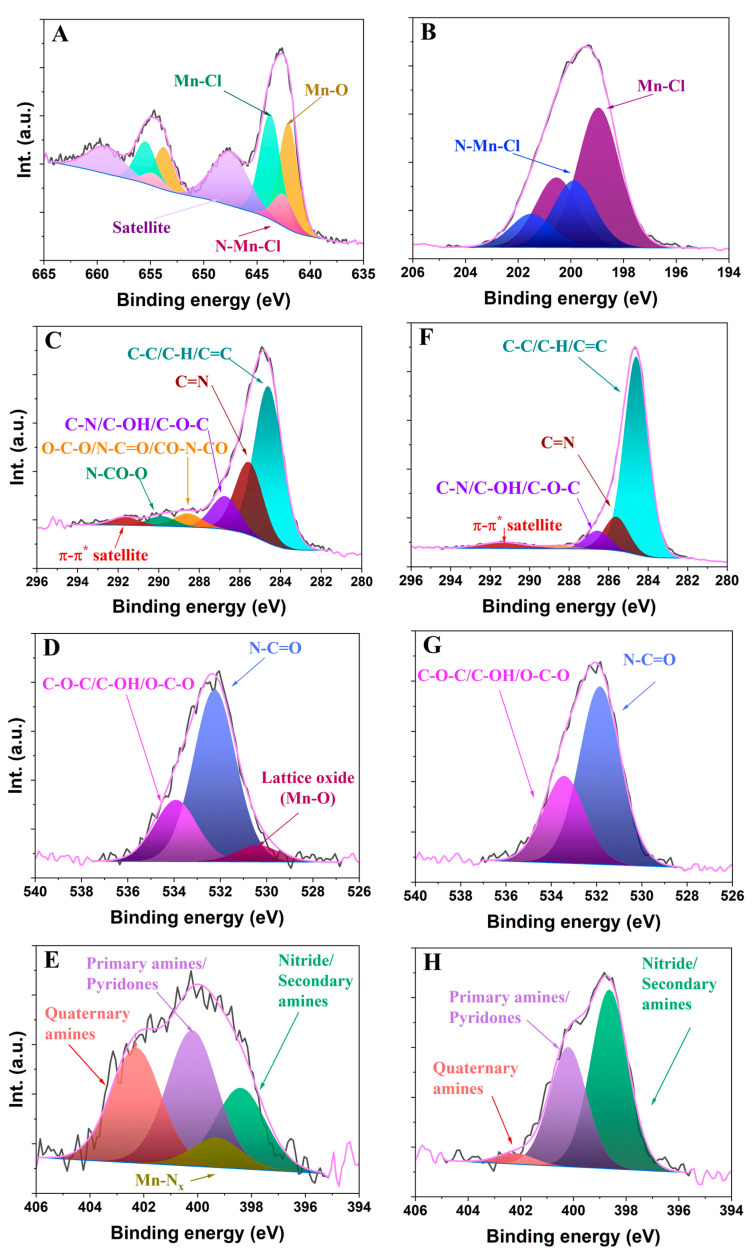
High-resolution XPS spectra of the Mn-CNDs (**A**) Mn 2p, (**B**) Cl 2p, (**C**) C1s,(**D**) O1s, (**E**) N1s and of the DPH-CNDs (**F**) C1s, (**G**) O1s, (**H**) N1s.

The composition of the carbon dots was further evaluated by XPS and EDX. Table 2 presents the elemental composition as determined by EDX (raw data is shown in Figure S4, Supplementary Information), compared to data obtained from XPS (Figure 2 shows the high-resolution core-level spectra of all the detected atoms; wide scan spectra are also shown in Figure S5) and flame atomic absorption spectroscopy (FAAS) for manganese. For comparison purposes, Table 2 also shows the composition of the Mn-doped carbon dots recalculated without chlorine (from the MnCl_2_ salt used to form the complex). The amount of bulk manganese detected by EDX and FAAS ranged between 4.8 and 5.8 at.%; the values are in line with the surface concentration of manganese of ca. 4.3 at.% detected by XPS. This confirms that a substantial amount of manganese has been effectively incorporated into the carbon dot structure.

The amounts of nitrogen (ca. 6–7 at.%) and oxygen (ca. 11.5–11.8 at.%) in the samples were rather high, which was expected based on the composition of the DPH ligand and the fact that the synthesis was carried out in an ambient (air) atmosphere. The amount of oxygen in the Mn-doped CD and the control without manganese was similar, which suggests a low contribution of oxidized manganese species (also confirmed by XRD and XPS; see discussions below). Other authors have reported similar observations for the preparation of manganese-doped CNDs through hydrothermal approaches with other precursors (citric acid, formamide, o-phenylenediamine) [30,31]. The good agreement between the values of elemental composition determined by bulk and surface techniques indicates the homogenous composition and distribution of the functionalization in the prepared materials (since XPS probes at a ca. 1–10 nm depth, and EDX analyzes depths from 1 to 3 μm) [32,33].

The analysis of the high-resolution XPS spectra showed differences among the samples upon the incorporation of manganese (Figure 2, Table 3). The Mn 2p spectrum of the Mn(II)-CNDs exhibits the two main peaks at 642.8 (Mn 2p_3_/_2_) and 654.5 (Mn 2p_1_/_2_) eV (2p^3^/_2_–2p^1^/_2_ splitting of 11.7 eV) and a satellite signal at 647.7 eV (≈29%) that confirm the presence of Mn (II) species. The deconvolution of the peaks shows two predominant contributions assigned to Mn-O (642.0 eV, ≈29%) and N-Mn-Cl moieties (643.8 eV, ≈32%), along with a small contribution of Mn-Cl coordination (642.5 eV, ≈10%), as also detected by EDX (Table 2). The position of these peaks is in line with the assignment reported in the literature [34,35]. The deconvolution of the C1s, O1s, and N1s core signals of the Mn(II)-CNDs and their control showed differences for the samples. The signal of N1s was deconvoluted in four main peaks at 398.5 eV attributed to nitride/secondary amines, 399.3 eV attributed to Mn-Nx, 400.2 eV assigned to primary amines/pyridines, and 402.3 eV associated with quaternary amine groups [34,36]. The main differences in the N1s profiles are the appearance of the Mn-Nx peak in the Mn-doped material assigned to the interactions between the nitrogen atoms and Mn ions and the increase in the relative contribution of the peak assigned to nitrogen in quaternary environments. Contributions of peaks at high binding energies related to oxidized nitrogen were not observed, despite the synthesis of the materials being carried out at moderate temperature under air atmosphere.

The high-resolution XPS spectrum of the C1s of the Mn-CNDs showed five main peaks at 284.6 eV (≈53%, attributed to aromatic sp^2^ carbon atoms), 285.6 eV (≈25%, assigned to C=N), 286.7 eV (≈11%, assigned to C-N/C-OH/C-O-C), 288.6 eV (≈5%, assigned to O-C-O/N-C=O/CO-N-CO), and 289.9 eV (≈3%, assigned to N-CO) [37,38,39,40]. A small broad band at 291.7 eV (≈3%) due to the π-π* satellite was also observed. The undoped sample displayed similar peaks, with a higher relative contribution of the peak at 284.6 eV (≈75%) and a lower relative contribution of the peak at 286.7 eV (C-N/C-OH/C-O-C, ≈7%). This indicates that the chemical environment of the bonds C-N, C-OH, C-O-C is influenced by the Mn atoms, in line with the Mn-Nx signal observed in the spectra of the N1s core level. A closer analysis of these results suggests that Mn-Nx moieties are most likely in a C–N–C configuration [37,38,39].

For the O1s, a peak assigned to Mn-O is observed in the Mn(II)-doped sample, with a decrease in the relative contribution of the peaks assigned to oxygen in single-bond moieties after the incorporation of manganese. The XPS high-resolution spectrum of chlorine revealed two main peaks associated with Mn-Cl (199.0–200.6 eV, ≈67%) from the MnCl_2_ precursor and N-Mn-Cl (199.9–201.5 eV, ≈33%) coordination [36]. All these confirm differences in the local chemical environment of oxygen and nitrogen atoms upon the incorporation of manganese in the material.

Differences were also observed in the surface charge distribution of the samples, as evidences by the zeta potential measurements (ca. −16.92 mV and −21.93 mV for DPH-CNDs and Mn-CNDs, respectively). The more negative zeta potential values observed for the Mn-doped sample suggest that amino groups present in the undoped material were either partially removed during synthesis or became coordinated to Mn^2+^ ions, as also proposed in the literature [34]. Other authors have reported a more positive zeta potential for Mn-doped CDs (−2.50 ± 0.98 mV) compared to Mn-free CDs (−12.04 ± 0.25 mV), which was attributed to the presence of positively charged Mn^2+^ ions on the surface [40]. The more negative values in our case imply that Mn^2+^ are distributed inside the carbon dot matrix rather than mainly localized on the surface.

The XRD patterns of the prepared samples (both DPH-CNDs and Mn(II)-CNDs) are shown in Figure S6. They show the typical broad peaks at 19.4° and 43.4° assigned to amorphous carbon materials with a turbostratic structure (see Figure S2) [41]. The narrow peaks between 16 and 22° in the diffractogram of the Mn-doped CNDs are attributed to the presence of crystalline structures (Table S1) assigned to Mn complexes through imidazole–nitrogen as bridging ligand in various configurations (PDF_02-088-0512, PDF_02-103-9616, PDF_05-008-2802).

**Figure 3:**
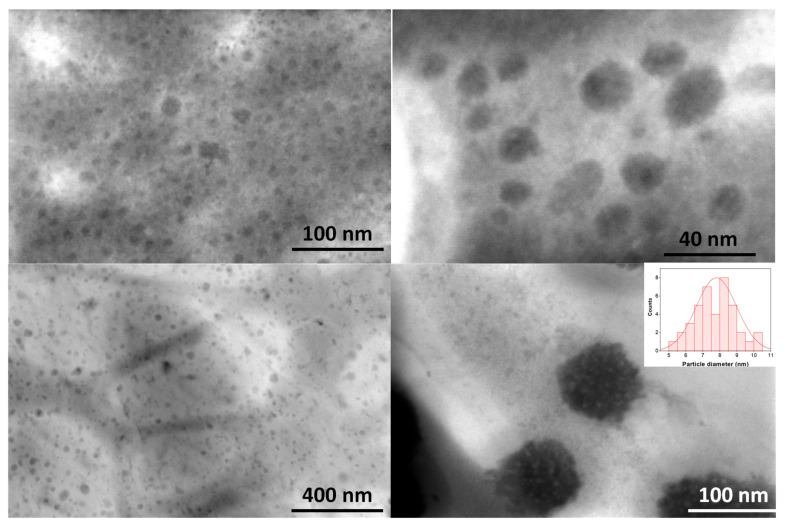
STEM micrographs of the Mn-CMDs suspended in ethanol (**top**) and water (**down**) at various magnifications. The inset represents the average size distribution of 40 Mn nanoparticles.

Figure 3 shows STEM micrographs of Mn-CDs dispersed in ethanol and water. The average particle size of the carbon dots depended on the dispersion solvent. The tendency of carbon dots (CDs) to form clusters upon interaction with different solvents has been widely reported in the literature [14,24,42]. The size of the aggregates was further confirmed by Dynamic Light Scattering (Figure S7), with two distinct particle size populations in water (42 and 197 nm) and water–ethanol mixtures (59 and 361 nm) and polydispersity indexes of 0.58 and 0.29, respectively. Figure 3 (down-right) also revealed that the particles exhibit a granular texture, which suggests a clustered organization of smaller nanostructures of approximately 7–8 nm in size (inset in Figure 3(down-right)).

The ethanolic suspensions of the CNDs displayed better stability over time than in water, with a monodispersed population of a 169 nm diameter consistently maintained throughout 2 h. Hence, to prevent any decrease in the photoluminescence of the materials due to particles clustering [14,24,42], the dispersions of Mn(II)-CNDs in ethanol were further used for the bioimaging applications.

### 2.2. Photoluminescence Properties of the Prepared CDs

**Figure 4:**
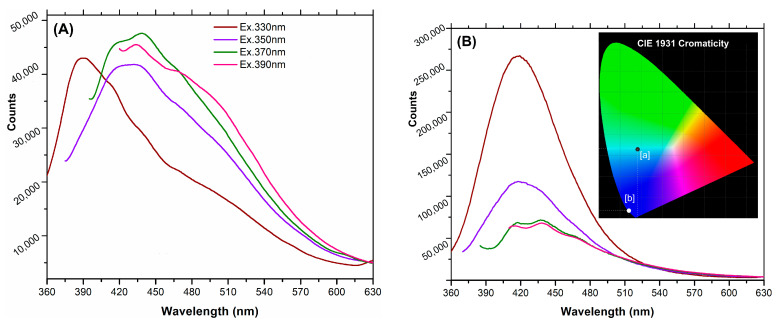
Emission spectra of (**A**) DPH-CNDs and (**B**) Mn(II)-CNDs recorded at various excitation wavelengths of 330, 350, 370, and 390 nm. Inset in plot (**b**) shows the chromaticity CIE 1931 chart of both samples.

Figure 4B shows the steady-state photoluminescence emission spectra of the Mn(II)-CND dispersions in water upon excitation within 310–400 nm (10 nm step). The Mn(II)-CNDs displayed a particularly bright emission with broad asymmetric bands (ca. 70–80 nm FWHM) with multiple emission peaks. This behavior is characteristic of samples with various fluorophores contributing to the emission profiles [43]. A markedly higher emission intensity was recorded for the Mn(II)-doped carbon dots compared to the undoped DPH-CND sample (Figure 4A). For the former, the most intense emission was achieved upon excitation at 370 nm (maximum emission at 438 nm), although differences were small among the four excitation wavelengths. In contrast, for Mn(II)-CNDs the highest emission intensity was obtained upon a 330 nm excitation (maximum at 419 nm), with two distinctive emission peaks (417–437 and 413–438 nm) upon excitation at 370 and 390 nm. For the undoped sample, a redshift in the position of the emission peak was observed for excitation above 330 nm. Such excitation-dependent emission behavior is commonly described for carbon dots. According to the literature, excitation above 320 nm involves transitions in surface fluorophores on the carbon dots (as opposed to those in the condensed aromatic carbon core) [14,43,44].

Table 4 compiles the absolute photoluminescence quantum yield (PLQY) of the Mn(II)-CNDs at four excitation wavelengths. The data showed different trends with the excitation wavelengths. The highest PLQY of 28.30% was obtained for the Mn(II)-CNDs upon excitation at 330 nm, being a value ca. four times higher than that of the undoped DPH-CNDs at the same excitation. While the PLQY of the Mn(II)-CNDs decreased as the excitation wavelength increased, the opposed trend was observed for the undoped DPH-CNDs. For the latter, the highest PLQY of 10.28% was recorded at a 390 nm excitation. These values of PLQY are similar to (and higher than) those reported in the literature for other N-doped carbon dots [45,46,47,48]. The chromaticity characteristics of both samples are also different (Figure 4b inset), with a perceived emission in deep blue for Mn(II)-CNDs (marked as [a] in the CIE 1931 chromaticity chart; x, y coordinates of 0.1872, 0.3456), compared to the blue-green region for the undoped sample (marked as [b] in the chromaticity CIE 1931 chart, x, y coordinates of 0.1545, 0.047). This is attributed to the presence of metallic ions in various structural configurations.

The lifetimes of the excited states of the Mn(II)-CNDs were measured both in water and EtOH dispersions (Table 5). The decay curves recorded at the 413 and 438 nm emission peaks are shown in Figure S8. Overall, the lifetimes were within the nanosecond range, which is typical for carbon dots [14,24,49]. In each measured condition, three different lifetimes were recorded due to the various emission species within the CNDs’ structure. In case of the Mn(II)-CNDs dispersed in water, the main contribution (~75%) is due to the species responsible for longer-lifetime (~12 ns) radiative processes, while in the case of the ethanol-dispersed Mn-CNDs the main contribution (32%) is achieved at 6 ns lifetimes. However, due to the specific conditions of each dispersion medium the contribution of various emissive species is different. The typical lifetimes reported for undoped carbon dots lie within the 4–6 ns range [14,24,49,50].

### 2.3. Biocompatibility and Cytotoxicity

**Figure 5:**
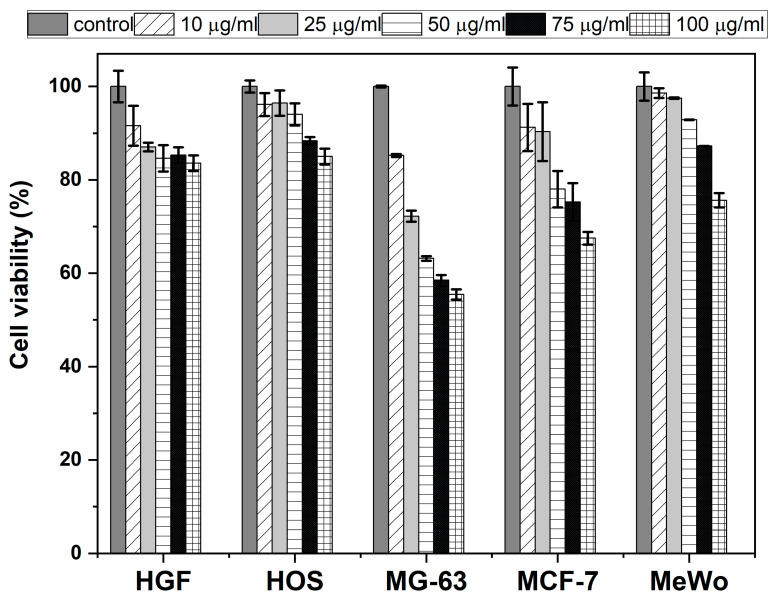
Cell viability of HGF, HOS, MG-63, MCF-7 and MeWo cells after 24 h incubation with different concentrations of the Mn-CNDs ranging between 10 and 100 µg/mL. The control of the untreated cells is also included for clarity. Data represent average values of three measurements and their respective standard deviation as error bars.

The biocompatibility and cytotoxicity of the synthesized carbon dots was evaluated by in vitro assays on both healthy and cancer cell lines. Gingival fibroblast (HGF) cells were chosen as the healthy cell control for assessing the biocompatibility, while various cancer cell lines were chosen for assessing the cytotoxicity, namely osteosarcoma (HOS, MG-63), breast adenocarcinoma (MCF-7), and malignant melanoma (MeWo) cell lines. The obtained data (Figure 5) evidences the overall good biocompatibility of the Mn(II)-CNDs to HGF cells, with only a slightly decrease in the cell viability (>84%) at all the tested concentrations. Similar studies on the cytotoxicity of carbon dots on HGF cell lines typically report viabilities above 90% for concentrations of carbon dots lower than those herein reported [51,52,53], indicating the large range of biocompatibility of our materials (even at high concentrations). On the other hand, large differences in cytotoxicity were observed for the studied cancer cell lines. No significant cytotoxic effects of the Mn(II)-CNDs were observed for HOS (viability > 85%) and MeWo (viability > 76%) cancer cell lines. In contrast, the Mn(II)-CNDs showed a somewhat cytotoxic effect for breast adenocarcinoma MCF-7 cells at 100 µg/mL (67% cell viability). The effect was more pronounced for MG-63 osteosarcoma cells, where a sharp decrease in the cell viability was observed at concentrations higher than 25 µg/mL. According to the literature, the cytotoxic effects of manganese-derived complexes in breast (MCF7) and bone (MG-63) cancer lines have been associated with the generation of radical oxygen species (ROS) that interfere with several cell functions (e.g., mitochondrial function, certain antioxidant enzyme activities) and induce the apoptosis of tumor cells [54,55,56,57,58,59]. Hence the strong cytotoxic effect observed for both cell lines would suggest the ability of the prepared Mn-CDs to produce ROS. The lower cytotoxicity of HOS and MeWo may be related to the higher resistance of these cell lines to ROS-induced effects. The different cytotoxic trend for HOS and MG63 (both bone cancer lines) may be explained by the lower stability of MG63 to ROS compared to HOS and further differences in morphology and functional properties that could alter the adhesion (thus interaction with the contrast agent) [60].

### 2.4. Fluorescence and Magnetic Resonance Imaging Applications of the Mn(II)-CNDs

Based on the bright luminescence of the carbon dot suspensions and their biocompatibility, we have explored their potential application as fluorescence and MRI probes. As discussed above, ethanolic suspensions of the Mn(II)-CNDs were used for bioimaging applications due to their stability over time and a stable average particle size in the colloidal suspensions. While this is not expected to have a large impact on the magnetic resonance response [61,62], it is well known that the clustering of nanoparticles can decrease the photoluminescence of the carbon dots due to internal reabsorption of the emitted light-shielding effects [14,24,42].

**Figure 6:**
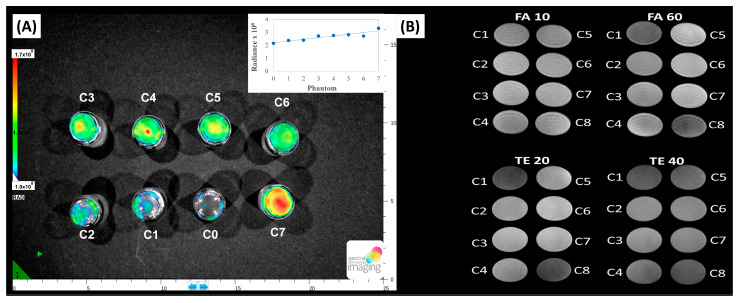
(**A**) In vitro fluorescence image of phantoms with increasing concentrations of the carbon dot suspensions. Excitation at 465 nm; emission at 530 nm; inset represents the evolution of the fluorescence intensity for the phantoms with increasing concentrations (from control C0 to C7); (**B**) T1 GRE (upper row) and T2 FSE (bottom row) magnetic resonance images of Mn(II)-CNDs at increasing concentrations from 0.005 to 0.2 mg/mL (C1–C7); C8 corresponds to the control of agarose gel.

Figure 6A shows the in vitro fluorescence optical imaging of phantoms with increasing concentrations of the carbon dots. As seen, the phantoms with the carbon dot suspensions presented a bright fluorescence emission (recorded at 530 nm; seen as green and red colors in the images) under excitation at 465 nm. The control in the absence of carbon dots (phantom C0) showed a poor fluorescence. The intensity of the fluorescence emission signal gradually increased with the amount of carbon dots in the dispersions, and the intensity of the signal was the same as that reported for other fluorescence probes [63,64]. These results demonstrate the great potential of the prepared carbon dots as contrast agents in imaging applications.

To assess the potential of DPH-Mn as a T1 and/or T2 contrast agent, the longitudinal (*r*_1_) and transverse (*r*_2_) relaxivity values were determined by applying specific equations to the average signal extracted from images acquired at different concentrations of the compound dispersed in 1% agarose gel [65,66,67]. Relaxation times were recorded at 20 °C and a physiological pH of 7.4 to simulate normal tissue conditions. Figure 6B presents the magnetic resonance phantom images of the plates. Relaxivity (R) is defined as the reciprocal of the relaxation time (1/T) per unit ion concentration, and it is determined as the slope of the curve as a function of Mn concentration. The data in Figure 6B is expressed as mM of Mn(II), recalculated from the content of manganese in the sample as determined by EDX (Table 2). This ensures consistency with published research on MRI contrast agents’ efficiency—although it should be noted that comparison should be made at similar magnetic fields, since the intensity of the MIR signal depends on this parameter, with higher values for smaller magnetic fields. As observed in the T1 GRE sequence (upper row), at small flip angles (e.g., FA 10°) no significant contrast differences were visible among the wells with different Mn(II)-CNDs concentrations. At a larger flip angle of 60°, the brightness increased with the concentration of manganese in the dispersion (a brighter response in the C7 well), while the control well of agarose gel (C8) and the wells with small amounts of Mn(II)-CNDs (e.g., C1) appeared darker. For the T2 FSE sequences (a constant flip angle of 90°) the opposed trend was observed; the best contrast response was obtained for an echo time of 20 ms, with an increased dark contrast with the concentration of manganese. The *r*_1_ and *r*_2_ relaxivity values represent the efficiency of the contrast agent in T1w and T2w sequences, respectively (specifically T1 GRE and T2 FSE in this study). The representation of R1 (1/T1) and R2 (1/T2) as a function of Mn(II) concentration showed that the relaxation times (T1 and T2) progressively decreased up to a concentration of 0.364 mM Mn(II) (0.08 mg/mL), while R1 and R2 increased (Figure S9). At concentrations of 0.45 and 0.9 mM Mn(II) (0.1 and 0.2 mg/mL), both R1 and R2 decreased, with a more pronounced effect in the T1 GRE mode. This indicates that the last two concentrations fall outside the linear range of relaxation time dependence on concentration and were therefore excluded from further analysis. Consequently, the dependence of R1 and R2 on the concentration of manganese was further analyzed in the range 0.023–0.364 mM Mn(II) (0.005–0.08 mg/mL).

The linear fitting of the data yielded relaxivity values of 0.932 s^−1^·mM^−1^ for *r*_1_ and 20.511 s^−1^·mM^−1^ for *r*_2_. Table 6 shows a comparison of these relaxivity values with those of contrast agents currently used in clinical practice, including gadolinium-based benchmarks. It is evident that while Mn(II)-CNDs exhibits a modest *r*_1_ value, its *r*_2_ value is significantly higher than that of commercial contrast agents based on gadolinium and manganese, which have a maximum reported *r*_2_ of 7.6 mM^−1^·s^−1^. However, it should be mentioned that such a comparison is not straightforward due to the differences in the applied magnetic field strengths (Table 6), as relaxivity values significantly depend on several acquisition parameters. Relaxivity values also depend strongly on the amount of metal ions of the samples [47]. A more adequate comparison with other metal-doped carbon dots reported in our previous studies under similar experimental conditions is also included in Table 6 [24].

**Figure 7:**
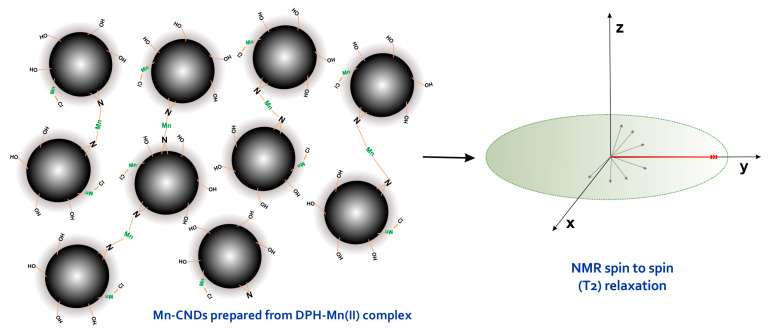
Schematized influence of the prepared Mn(II)-CNDs over the spin to spin (T_2_) relaxation.

The high *r*_2_ value indicates that spin–spin relaxation effects are predominant in the prepared Mn(II)-CNDs. This points out that the sample is a good T2-weighted (negative) contrast agent, as further confirmed by the relaxivity ratio *r*_2_/*r*_1_ of ca. 22—according to the literature, the *r*_2_/*r*_1_ ratio defines the type of contrast agent, with values up to 5 for positive or T1-weighted and ratios above 10 for negative or T2-weighted contrast agents [67]. We attribute this behavior to the nature of the manganese species in the prepared carbon dots. Indeed, data from XPS and EDX confirmed that manganese is incorporated as Mn(II) ions—either in a Mn-O or Mn-Nx configuration—as higher oxidation states of manganese were not detected. This assures a favorable density of spins (d5 configuration) for MRI applications. Also, the bulk and surface elements composition (Table 2) confirmed that Mn(II) is predominantly located in the external surface of the carbon dots. This environment provides suitable conditions for a fast proton/water exchange, responsible for a strong MRI contrast signal mainly by shortening the transverse relaxation time (T2) to produce dark images (Figure 7). A similar phenomenon has been observed for other superparamagnetic iron oxides acting as negative contrast agents [62].

## 3. Materials and Methods

### 3.1. Materials

5,5′-Diphenylhydantoin (DPH, 99%) and MnCl2 (99%) were supplied by Merck Chemicals. Ultra-pure distilled water (Millipore-Direct Q) and ethanol (EtOH, 97%) were used in the synthesis.

### 3.2. Synthesis of the Mn-Doped Carbon Dots

In the first stage, the Mn(II)-DPH complex with a 1:2 metal to ligand ratio was synthesized. In a typical procedure, the adequate amount of DPH is dissolved under stirring in a 1:1 *v*/*v* H_2_O/EtOH mixture and then mixed with the appropriate amount of MnCl_2_ to reach 1:2 metal–ligand ratio and stirred until the complete dissolution of the manganese salt occurs. Afterwards, the solution containing DPH and MnCl_2_ is transferred to a flask with a reflux condenser and heated at 85 °C for 24 h to allow complexation as indicated in the following reaction:

2C_15_H_12_N_2_O_2_ + MnCl_2_ → [Mn(C_15_H_11_N_2_O_2_)_2_] + 2HCl↑

A pale brown precipitate was recovered by centrifugation at 5000 rpm for 5 min and washed at least three times in distilled water. The purified precipitate of the DPH-Mn(II) complex was dried in vacuum for about 24 h at 80 °C. In the second stage, the DPH-Mn(II) complex powders were thermally processed in a quartz-made reactor equipped with a temperature/flow regulated hot air gun, as described elsewhere [15,25]. In a typical synthesis, about 0.25 g of DPH-Mn(II) complex is heated under atmospheric conditions at 470–490 °C for 10–11 min. Afterwards, the solid remaining in the quartz tube is flooded in 10 mL of cold water (ca. 5 °C). The dispersion was centrifuged at 10,000 rpm for 10 min to remove the large particles, followed by a final filtration stage with a 0.22 μm membrane. The clear transparent aqueous suspension with the Mn(II)-CNDs was collected and freeze-dried until further use. For STEM analysis, cold ethanol was used as solvent in the flooding step (instead of water).

### 3.3. Characterization Techniques

Infrared spectra of the samples were recorded in a Shimadzu IRAffinity 1S spectrometer. The dried samples were pressed into KBr pellets (dilution 1:1000). Each spectrum resulted from the accumulation of 256 scans, recorded with a spectral resolution of 1 cm^−1^ in the 4000−400 cm^−1^ spectral domain. Thermogravimetric analyses of the samples were recorded in a thermobalance (Netzsch- STA 449 F1 Jupiter) under an inert atmosphere, with a heating rate of 10 °C/min within 30–700 °C. X-ray photoelectronic spectroscopy (XPS) spectra of the dried samples were collected in a ULVAC-PHI, 5000 VersaProbe spectrometer (physical Electronics) using AlKα radiation (1486.7 eV) operating at 20 mA and 15 kV and with a pass energy of 20 eV and a step size of 0.1 eV. Processing of the high-resolution XPS spectra was carried out in CasaXPS (version 2.3.23PR1.0) with the C1s peak of adventitious carbon at 284.6 eV as a reference signal. The binding energy values were accurate within ±0.2 eV. The dimensional analysis, zeta potential (ƺ), and particle size distribution of aqueous dispersions of the samples were recorded on a Zetasizer Advance Pro Red instrument. Prior to the measurements, the solutions were centrifuged twice at 15,000 RPM for 10 min. The concentration of manganese in the carbon dots was determined by flame atomic absorption spectroscopy (FAAS). The detailed methodology is provided in the Supplemental Information. The X-ray diffraction (XRD) patterns of the samples were measured with a Rigaku SmartLab X-ray diffractometer in Bragg–Bretano geometry using a Cu anode (X-ray wavelength of 1.5406 Å). The morphology of the samples was analyzed in a Verios G4 UC scanning electron microscope (Thermo Scientific) equipped with an energy-dispersive X-ray spectroscopy (EDX) analyzer (Octane Elect Super SDD detector, Pleasanton, CA, USA). Scanning transmission electron microscopy (STEM) studies were conducted with the STEM 3+ detector in Bright-Field mode, operating at an accelerating voltage of 25 kV and a spot size of 0.1 nA. The suspensions of the samples were ultrasonicated and deposited on carbon-coated copper grids with a 300-mesh size and air-dried for 24 h in dust-free conditions at ambient temperature. EDX spectra were recorded at an accelerating voltage of 25 kV and a spot size of 6.4 nA. The steady-state fluorescence of freshly prepared dispersions of the samples was measured in a FluoroMax 4P spectrophotometer (Horiba, Tokyo, Japan) at excitation wavelengths between 300 and 400 nm, in quartz cuvettes with a path length of 10 mm (excitation and emission slits of 5 nm). The excitation and emission slits were set at 2 nm. The absolute photoluminescence quantum yield (PLQY) was recorded with a Quanta Φ integration sphere module, using FluorEssence software (ver. 3.5.1.20) for the spectral acquisition and the calculation of the PLQY and CIE1931 chromaticity parameters. Excited states lifetimes (LTs) were recorded in a FluoroHub Time-Correlated Single-Photon Counting lifetime module with a 370 nm LED excitation source. Experimental data was fitted to a single or multi-exponential decay model, based on each sample’s behavior.

### 3.4. Cell Viability Assay

The biocompatibility and cytotoxicity of the Mn-CNDs were assessed with the CellTiter-Glo^®^ 2.0 Assay (Promega, Madison, WI, USA), according to the manufacturer’s instructions. Cells were cultured in complete cell culture medium: αMEM medium with 10% fetal bovine serum and 1% antibiotic–antimycotic (Penicillin-Streptomycin–Amphotericin B mixture, all from Gibco, Thermo Fisher Scientific, Waltham, MA, USA). A human gingival fibroblast (HGF) cell line was selected to assess the biocompatibility of the prepared materials. Several cancer cell lines have been used to evaluate their cytotoxicity, namely osteosarcoma (HOS, MG-63), breast adenocarcinoma (MCF-7), and malignant melanoma (MeWo) cell lines (all from CLS Cell Lines Service GmbH, Eppelheim, Germany), covering most common types of cancer (breast, skin, bones). The cells were seeded separately into 96-well white opaque tissue culture-treated plates (50,000 cells/mL–HGF and 100 000 cells/mL–cancer cell lines) in complete cell culture medium and allowed to adhere overnight. The next day, the cells were incubated in triplicate experiments with different concentrations of Mn(II)-CNDs (10, 25, 50, 75, 100 µg/mL) for 24 h, following the recommendation outlined in ISO 10993–5:2009 (E) [27]. For the determination of cell viability, CellTiter-Glo^®^ reagent was ad nd luminescence was recorded in a ded, a FLUOstar^®^ Omega V6.20 microplate reader (BMG LABTECH, Ortenberg, Germany). Treated cells’ viability was calculated as percentage of untreated cells’ viability, and data were represented graphically as means ± standard error of the mean.

### 3.5. In Vitro Fluorescence Imaging

In vitro fluorescence imaging was recorded in a SPECTRAL Ami HT instrument piloted by Aura imagingV.4.0 software. About 1 mL of carbon dot suspensions of varied concentrations was deposited in 5 mL glass phantoms. The imaging procedure included excitation at 430 nm (LED power 60%), emission at 530 nm, an exposure time of 1 s, FOV 25 × 17.5 cm, F-stop 2, and binning 2. The quantification of the fluorescence was conducted over circular regions of interest, covering the whole image of each sample.

### 3.6. In Vitro Magnetic Resonance Imaging (MRI) Assays

The potential application of the prepared materials as MRI contrast agents was evaluated in in vitro cell/tissue cultures of 1% agarose gel prepared in 0.01 M PBS (pH 7.4) to determine values of longitudinal (r1) and transversal (r2) relaxivities, calculated from the respective T1 and T2 relaxation times. Agarose not only mimics a cellular culture environment but also prevents compound sedimentation upon scanning, which could otherwise significantly impact the results. The prepared carbon dot suspensions were dispersed in this medium as indicated elsewhere [24,28]. Briefly, a stock solution of 2 mg/mL of the CNDs in ultrapure water was prepared by ultrasonication for 30 min. Defined volumes of this solution were subsequently added to the hot 1% agarose solution to achieve concentrations in the range of 0.01–0.4 mg/mL. The solutions were homogenized in their preparation vials and then transferred in 3 mL aliquots into a well plate (wells C1–C7) until complete solidification. A control sample (well C8) was filled with 3 mL of agarose gel without the CNDs. MRI scanning of the well plates was performed in nanoScan PET-MRI equipment provided with a magnetic field strength of 1 Tesla, employing standard T1-weighted (T1w) and T2-weighted (T2w) sequences, specifically T1 gradient echo (GRE) and T2 fast spin echo (FSE), with B0 magnetic field shimming and coil calibration at water proton frequency. The plate of the samples was positioned horizontally at the center of the coil’s field of view. The main parameters for the T1 GRE imaging acquisition were as follows: the repetition time TR 360 ms, the echo time TE 3.8 ms, the number of excitations NSA 2, slice thickness 3 mm, slice gap 1 mm, and the flip angle (FA) variable (ca. 10, 20, 60, 70°). The T2 FSE acquisition parameters were TR 1895 ms, NSA 2, slice thickness 3 mm, FA 90° with a variable TE (ca. 20, 40, 80, and 120 ms). On the reconstructed MR images, circular regions of interest (ROIs) of 10 mm diameter were drawn inside each sample; a representative slice placed ca. in the middle of the agarose gel volume was selected to assure a uniform MRI signal. T1 relaxation times were determined by a two-point estimation method for the flip angles of 10 and 60° [29], using the formula

$$\ln\left[\frac{\left(I_{1}sin\theta_{2}-I_{2}sin\theta_{1}\right)}{\left(I_{1}sin\theta_{2}cos\theta_{1}-I_{1}sin\theta_{2}cos\theta_{1}\right)}\right]=\frac{-TR}{T_{1}}$$

where I_1_, I_2_ are the mean signal intensities measured inside the regions of interest of the samples at 10 and 60 ° flip angles; TR is the repetition time; θ_1_ and θ_2_ are the flip angles.

T_2_ relaxation times were obtained from the 1H relaxometry tool of Nucline softwareV1.02, using the equation

$$I=A\cdot e^{\frac{TE}{T2}}$$

where I is the mean signal intensity in the ROI, A is the initial intensity of the signal, and TE is the echo time.

Examples of the graphical calculations of T_1_ and T_2_ are shown in the Supplementary Information File (Figures S1–S3).

## 4. Conclusions

In this study we report the preparation of Mn-doped carbon dots prepared by the thermal decomposition at a mild temperature of a Diphenylhydantoin–Mn(II) complex, and their use as a magnetic resonance imaging contrast agent. The choice of the Diphenylhydantoin–Mn(II) complex as a precursor for the preparation of Mn(II)-doped carbon dots has proven to be a good approach to obtain a good dispersion of the metallic cations in the matrix of the carbon nanodots. The Mn(II)-CNDs displayed photoluminescence features with an intense emission in the blue region, which suggests their potential application as a fluorescence marker in biology-related investigations. The cytotoxicity assays revealed the good biocompatibility of the Mn(II)-CNDs on normal gingival fibroblast and malignant melanoma cell lines, with cell viabilities above 80–90% for concentrations of carbon dots higher than those reported in the literature for other nanostructures. In contrast, the Mn(II)-CNDs showed a cytotoxic effect on osteosarcoma (MG-63) and breast adenocarcinoma (MCF-7) cells lines at concentrations above 25 µg/mL. The in vitro magnetic resonance imaging assays rendered higher r_2_ relaxivity values for the Mn(II)-CNDs than those reported for commercial gadolinium-based contrast agents. In sum, the simplicity of the synthetic pathway allows a good dispersion of the metallic ions in the carbon dots matrix, along with a low cytotoxicity. As a result, the Mn(II)-CNDs prepared from a complex precursor have the potential to become a viable alternative as T2-weighted contrast agents, offering more accurate MRI signals in combination with T1-weighted contrast agents (e.g., based on gadolinium complexes) in biomedical applications. The next steps should focus on the optimization of their performance under different magnetic field conditions and the assessment of their biocompatibility for clinical applications in in vivo assays.

## Figures and Tables

**Table 1:** Summary of main IR bands and assignment to specific vibration groups recorded for DPH ligand, Mn(II)-DPH complex, and Mn-CNDs.

Description	Recorded Peaks (cm^−1^)		
	DPH	Mn(II)-DPH	Mn(II)-CNDs
N-H stretch	3479	-	-
OH stretch	3273	3274	included in 3279/3210 broad peak
C-H stretch	3070	-	included in 3279/3210 broad peak
C=C/C-C stretch	2799/2711/1235	2709/2703	included in 3279/3210 broad peak
C=O asym. stretch	1774	1772	included in 1610 broad peak
C=O sym. stretch	1717	1718	included in 1610 broad peak
Amide band	-	-	included in 1610, 1529 broad peaks
C-N-H deformation	1491	1491	1451
Aromatic ring stretch	1445	1445	included in broad peak of 1610
N-H def.	1403	1404	-
N-C=O def.	749/696	749/697	760/695
C-N-C def.	647	646	612
C-C=O def.	519	518	512

**Table 2:** Elemental composition (at.%) of samples DPH-CNDs (undoped) and Mn(II)-CNDs recorded by EDX, XPS, and FAAS.

Element	EDX			XPS			FAAS
	DPH-CNDs	Mn(II)-CNDs	Mn(II)-CNDs Normalized Without Cl	DPH-CNDs	Mn(II)-CNDs	Mn(II)-CNDs Normalized Without Cl	Mn(II)-CNDs
C	90.81	70.32	76.14	86.84	53.06	75.41	-
N	7.71	5.63	6.10	7.07	4.91	6.95	-
O	1.48	10.60	11.48	6.09	8.34	11.81	-
Mn	-	5.81	6.29	-	4.30	6.09	4.83
Cl	-	7.64	-	-	29.40	-	

**Table 3:** Relative abundance distribution (%) and position (eV) of the surface groups detected in sample Mn-CNDs and DPH-CNDs upon deconvolution of the high-resolution core-level XPS spectra.

C1s	Csp_2_ (284.6 eV)	C=N (285.6 eV)	C-N/ C-OH/ C-O-C (286.7 eV)	O-C-O/N-C=O/CO-N-CO (288.6 eV)	N-CO-O (289.9 eV)	π-π* (291.7 eV)
Mn-CNDs	53.25	24.75	11.28	4.58	3.35	2.79
DPH-CNDs	74.95	12.74	6.93	1.24	-	4.14
O 1s	Mn-O (530.2 eV)		N-C=O (532.1 eV)	C-O-C/C-OH/O-C-O (533.8 eV)		
Mn-CNDs	6.72		68.69	24.59		
DPH-CNDs	-		67.11	32.89		
N 1s	Nitride/ Secondary amines (398.5 eV)		Mn-Nx (399.3 eV)	Primary amines/ Pyridones (400.2 eV)		Quaternary amines (402.3 eV)
Mn-CNDs	22.36		8.28	37.52		31.84
DPH-CNDs	57.87		-	38.62		3.51
Mn 2p	Mn-O (642.0 eV)		Mn-Cl (642.5 eV)	Mn-Nx (643.8 eV, eV)		Satellite (647.7 eV)
Mn-CNDs	29.33		9.78	31.98		28.91
DPH-CNDs	-		-	-		-

**Table 4:** Absolute PLQY values for sample Mn(II)-CNDs and DPH-CNDs at various excitation wavelengths.

Excitation (nm)	330	350	370	390
DPH-CNDs	6.89	9.17	9.30	10.28
Mn(II)-CNDs	28.30	9.60	4.05	3.51

**Table 5:** Photoluminescence lifetime of the excited states recorded at various emission wavelengths for Mn-CNDs dispersed in H_2_O and EtOH.

Sample Code	τ_1_ (ns)	a_1_ (%)	τ_2_ (ns)	a_2_ (%)	τ_3_ (ns)	a_3_ (%)	<τ> (ns)	χ^2^
Mn-CNDs–water λ_em_ = 413 nm	12.2	78.5	4.3	17.4	0.82	4.1	11.6	1.05
Mn-CNDs–water λ_em_ = 438 nm	11.6	76.6	3.8	18.9	0.49	4.5	11.0	1.17
Mn-CNDs–ethanol λ_em_ = 413 nm	6.4	58.5	2.2	32.2	0.56	9.3	5.6	1.12
Mn-CNDs–ethanol λ_em_ = 438 nm	6.6	55.0	2.6	32.3	0.61	12.8	5.8	0.98

**Table 6:** Relaxivity values of the prepared Mn-CDs compared to commercial gadolinium-based contrast agents currently used in clinical practice and other Mn-doped and metal-doped CDs reported in the literature.

Material	*r*_1_ [mM^−1^·s^−1^]	*r*_2_ [mM^−1^·s^−1^]	Magnetic Field (T)	Reference
Gadovist (Gadobutrol) (in plasma, 37 °C)	5.2	6.1	1.5	[68]
Dotarem (Gadoterate Meglumine)	3.6	4.3	1.5	
ProHance (Gadoteridol)	4.1	5.0	1.5	
Magnevist (Gadopentetate Dimeglumine)	4.1	4.6	1.5	
MultiHance (Gadobenate Dimeglumine)	6.3	8.7	1.5	
Omniscan (Gadodiamide)	4.3	5.2	1.5	
Mn carbon dots (up to 1 at. Mn)	9.7/4.8/6.7	89/42/67	1.5	[31]
Mn carbon dots (0.1 mM Mn)	2.3	--	1.5	[47]
Mn_2_A_l1_-LDH	0.6	17.9	16.4	[69]
Mn_0.5_Mg_2.6_A_l1_-LDH	1.2	--	16.4	[28]
MnFe_2_O_4_@PEGa	0.7	118	9.4	[70]
MnO	0.5–0.1	1.7–0.4	3	[71]
MnOx–SiO_2_ hollow@PEG	0.8	27.7	3	[72]
MnO_2_@PEG	0.007	-	3	[73]
Mn_3_Fe_1_-LDH	0.08	--	0.5	[74]
MnSO_4_	3.8	19	1	[75]
Mn_CP	2.5	13	1	[28]
CU_CP	5.5	24	1	
FU_CP_IE	4	22	1	
Mn(II)-CNDs	1.0	21	1	this work
Material	*r*_1_ [mL mg^−1^·s^−1^]	*r*_2_ [mL mg^−1^·s^−1^]	magnetic field (T)	reference
Mn-CNDs-NHF	17.8	88.6	1	[24]
Fe-CNDs-NHF	0.2	48.7	1	
Gd-CNDs-NHF	10.0	22.1	1	
Mn(II)-CNDs	87.2	1541	1	this work

## Data Availability

The raw data supporting the conclusions of this article will be made available by the authors upon request.

## Supplementary Materials

The following supporting information can be downloaded at: https://www.mdpi.com/article/10.3390/ijms26136293/s1.
