# Supplementary material for: Mn-Doped Carbon Dots as Contrast Agents for Magnetic Resonance and Fluorescence Imaging
Source: Int J Mol Sci. 2025 Jun 29;26(13):6293. doi: 10.3390/ijms26136293 (PMC12249806; doi:10.3390/ijms26136293)
Supplement: Supplementary file 1 [file ijms-26-06293-s001.zip › ijms-3676777-supplementary.pdf]

(Supplementary Information File)

Flame atomic absorption spectroscopy (FAAS)

Raw data and graphical calculations of T1 and T2 relaxation times

Figure S1. Representative Magnetic Resonance images and circular regions of interest (ROI) of 10 mm diameter of the agarose gel cultures containing Mn-CND at flip angles between 10-70º used to determine relaxivities and T1 relaxation times as indicated in the formula above.

Figure S2. Representative Magnetic Resonance images and circular regions of interest (ROI) of 10 mm diameter of the agarose gel cultures containing Mn-CND at echo times (TE) between 20 and 120 used to determine the intensities of the signals for the calculation of the T2 relaxation times using the equation indicated above.

Figure S3. Example of T2 graphical analysis from Nucline software comprising the relaxation curve and additional parameters.

Table S1. Parameters of the main peaks detected in the XRD diffractograms of samples DPH-CND and Mn-CND.

Figure S4. EDX spectra of Mn-CNDs (A) and DPH-CNDs (B) samples.

Figure S5. Wide XPS spectra recorded for the prepared DPH-CNDs and Mn-CNDs.

Figure S6. Powder XRD diffractograms of DPH-CNDs with background correction (A), Mn-CNDs without background correction, with an insert showing the expanded diffractogram between 24-30^0 2θ (B) and Mn-CNDs with background correction (C).

Figure S7. DLS measurements of Mn-CNDs dispersed in ethanol (A), ethanol:water (1:1 v/v) (B), and water (C).

Figure S8. Photoluminescence lifetime decay recorded for the Mn-CNDs at 413 and 436 nm emission peaks.

Figure S9. The dependence of R1(1/T1) and R2(1/T2) on the concentration of Mn^{2+} ions.

Flame atomic absorption spectroscopy (FAAS)

The concentration of Mn was measured from solutions by flame atomic absorption spectroscopy (FAAS). The data were acquired using a Continuum Source Atomic Absorption Spectrometer - contrAA 300 (AnalitykJena, Germany) - equipped with an optimized high-resolution Echelle double monochromator. A device such as CCD line detector with high quantum efficiency and increased UV sensitivity can detect the amount of reduction of the light intensity due to absorption by the analyte, and this can be directly related to the amount of the element in the sample. The technique of FAAS requires a liquid sample to be aspirated, aerosolized, and mixed with combustible gases, such as acetylene and air or acetylene and nitrous oxide, depending of the element determination, we used Air-Acetylene mixture at a flow of 80L/h. The aerosol of the sample is produced in a pneumatic concentric jet nebulizer (Platinum/Rhodium tube, PEEK nozzle, PEEK cap). To provide element specific wavelengths, a light beam from a xenon short-arc lamp with UV arc passed through the hot spot having an automatic hot-spot tracking and simultaneous drift correction. The mixture is ignited in a flame whose temperature ranges from 2100 to 2800 °C. The wavelength range of FAAS measurements is of 190-900 nm and accuracy ensured through automatic neon wavelength correction, for Mn determination the wave length used is 279.4817 nm (Figure S7). The spectra were collected and as a function of the absorbance the element concentrations were calculated. The spectral band width is of 2 pm at 200 nm. The characteristic wavelengths are element specific and accurate to 0.01-0.1nm. The sample was aspirated for manganese (Mn) determination using Atomic Absorption Spectroscopy (AAS), with three replicates performed to ensure accuracy and reproducibility of the measurements. Following the analysis, the calculated Mn concentration in the sample of 0.531 mg/L Mn-CNDs was determined to be 1.41 mg Mn/L (approx. 0.0238 Mn atoms/L). This value represents the mean concentration obtained from the triplicate measurements, reflecting the precision of the analytical method employed.

Raw data and graphical calculations of T1 and T2 relaxation times

The potential application of the prepared materials as MRI contrast agents was evaluated using an in-vitro cell/tissue cultures of 1% agarose gel prepared in 0.01 M PBS (pH 7.4) to determine values of longitudinal (r1) and transversal (r2) relaxivities, calculated from the corresponding T1 and T2 relaxation times. Agarose not only mimics a cellular culture environment but also prevents compound sedimentation during scanning, which could otherwise significantly impact the results. The prepared carbon dots suspensions were dispersed in this medium as indicated elsewhere [24,28]. Briefly, a stock solution of 2 mg/mL of the CNDs was prepared by solubilizing the obtained powders in ultrapure water by ultrasonication for 30 minutes. Defined volumes of this solution were subsequently added to the hot 1% agarose solution to achieve concentrations in the range 0.01-0.4 mg/mL. The solutions were homogenized in their preparation vials and then transferred in 3 mL aliquots into a well plate (wells C1-C7) until complete solidification. A control sample (well C8) was filled with 3 mL of agarose gel without the CNDs. MRI scanning of the well plates was performed using a nanoScan PET-MRI equipment provided with a magnetic field strength of 1 Tesla, employing standard T1-weighted (T1w) and T2-weighted (T2w) sequences, specifically T1 gradient echo (GRE) and T2 fast spin echo (FSE), with B0 magnetic field shimming and coil calibration at a water proton frequency. The plate containing the samples to be analyzed was positioned horizontally at the center of the coil's field of view. The main parameters for T1 GRE imaging acquisition were: the repetition time TR 360 ms, the echo time TE 3.8 ms, the number of excitations NSA 2, slice thickness 3 mm, slice gap 1 mm and the flip angle (FA) was variable (ca. 10, 20, 60, 70º). The T2 FSE acquisition parameters were: TR 1895 ms, NSA 2, slice thickness 3 mm, FA 90º with a variable TE (ca. 20, 40, 80 and 120 ms). On the reconstructed MR images, circular regions of interest (ROI) of 10 mm diameter were drawn inside each sample; a representative slice placed ca. in the middle of the agarose gel volume was selected to assure a uniform MRI signal. T1 relaxation times were determined by a two-point estimation method for the flip angles of 10 and 60º [29], using the following formula:

$$\ln\left[\frac{(I_1 sin\theta_2 - I_2 sin\theta_1)}{(I_1 sin\theta_2 cos\theta_1 - I_1 sin\theta_2 cos\theta_1)}\right] = \frac{-TR}{T_1}$$

where I1, I2 are the mean signal intensities measured inside the regions of interest of the samples at 10 and 60 ° flip angles; TR is the repetition time; θ1 and θ2 are the flip angles.

T2 relaxation times were obtained from the 1H relaxometry tool of Nucline software, using the equation:

$$I = A \cdot e^{\frac{TE}{T2}}$$

where I is the mean signal intensity in the ROI, A is the initial intensity of the signal, and TE is the echo time. Examples of the graphical calculations of T1 and T2 are shown in the Supplementary Information File.

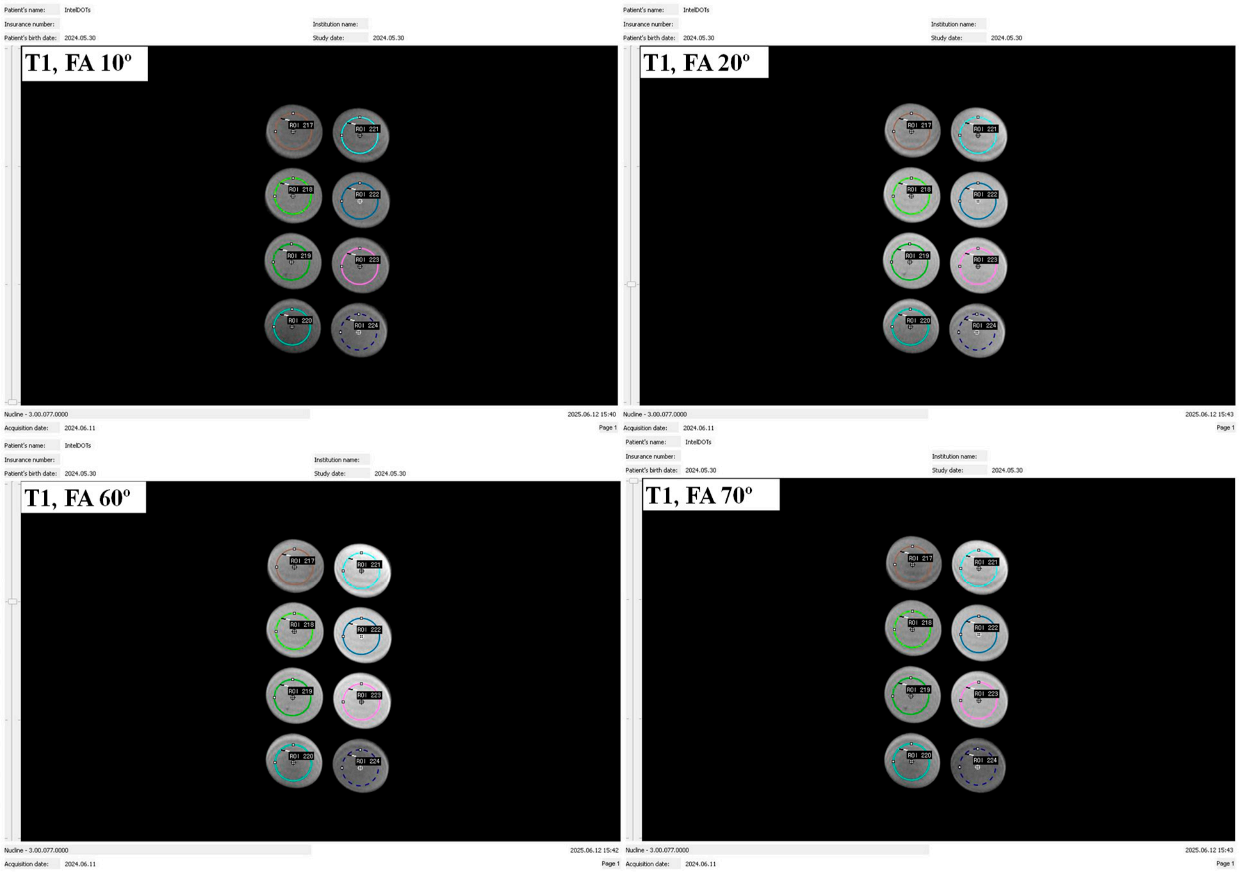

Figure S1. Representative Magnetic Resonance images and circular regions of interest (ROI) of 10 mm diameter of the agarose gel cultures containing Mn-CND at flip angles between 10-70º used to determine relaxivities and T1 relaxation times as indicated in the formula above.

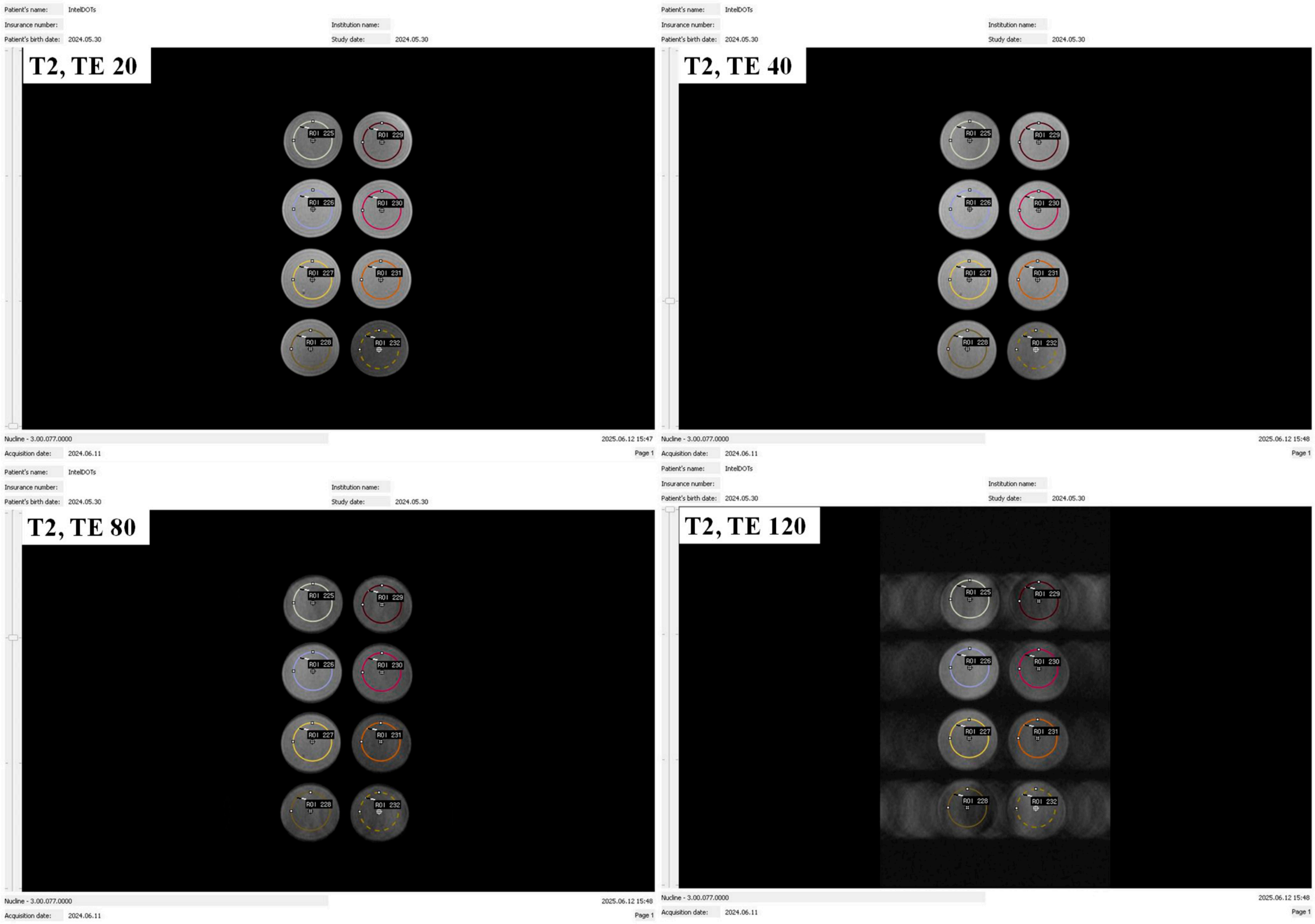

Figure S2. Representative Magnetic Resonance images and circular regions of interest (ROI) of 10 mm diameter of the agarose gel cultures containing Mn-CND at echo times (TE) between 20 and 120 used to determine the intensities of the signals for the calculation of the T2 relaxation times using the equation indicated above.

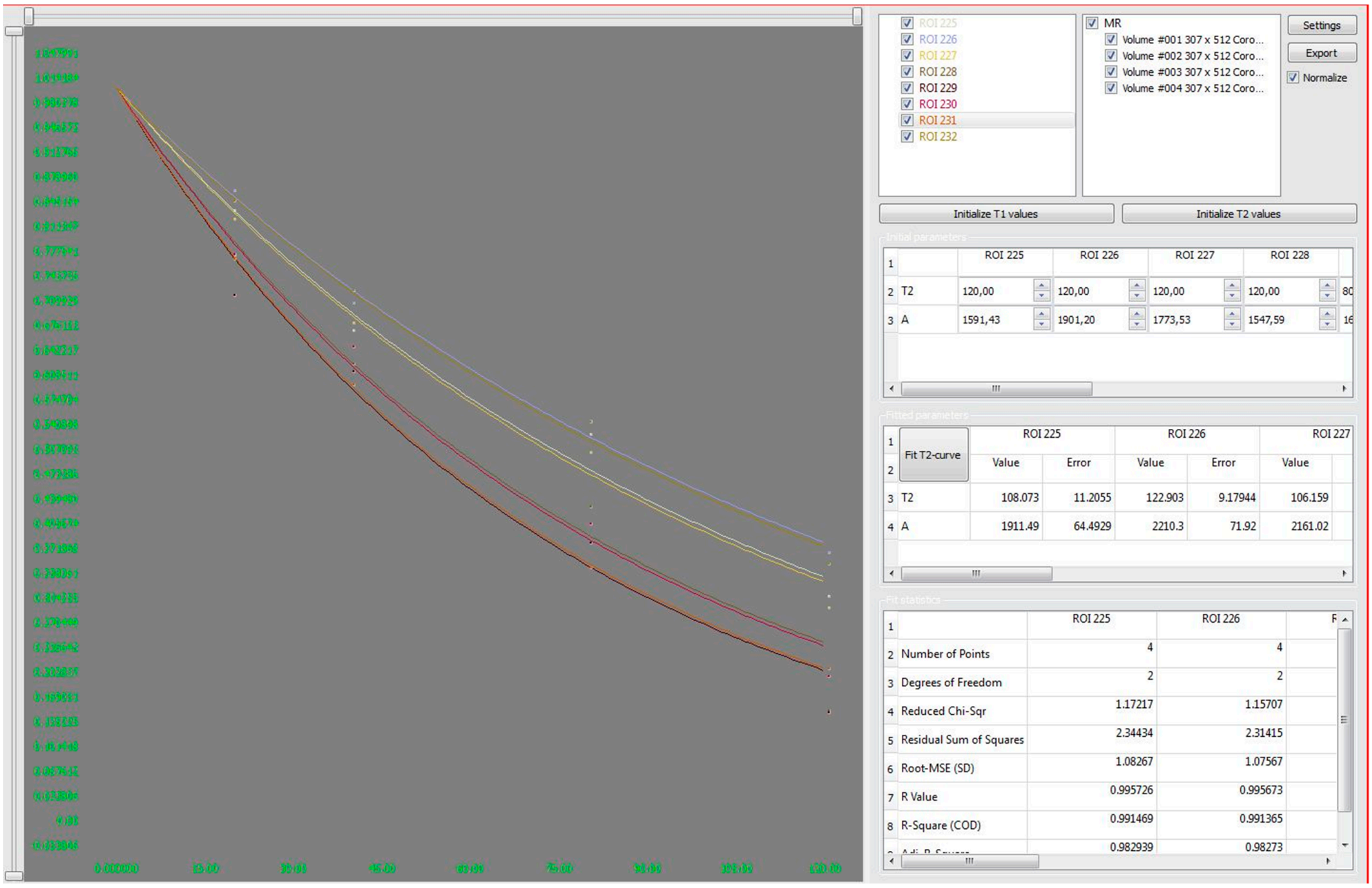

Figure S3. Example of T2 graphical analysis from Nucline software, comprising the relaxation curve and additional paramters

,

Table S1. Parameters of the main peaks detected in the XRD diffractograms of samples DPH-CND and Mn-CND.

**Sample DPH-CND**								
Peak No.	2θ (°)	e.s.d.	d* (Å)	e.s.d**.	Height, (cps)	e.s.d.	FWHM*** (°)	e.s.d.
1	4,818	0,01224	18,327	0,0465	18887,7	153,8	6,6	0,0259
2	19,387	0,0212	4,575	0,0045	10775,9	99,4	10,7	0,0610
3	43,427	0,0767	2,082	0,0035	2003,4	46,2	17,3	0,4355
**Sample Mn-CND**								
Peak No.	2θ (°)	e.s.d.	d* (Å)	e.s.d**.	Height, (cps)	e.s.d.	FWHM*** (°)	
1	11,422	0,0141	7,741	0,0095	297,9	11,7	0,0971	
2	12,385	0,2656	7,141	0,1526	485,6	23,6	10,5394	
3	16,672	0,007	5,313	0,0022	418,2	17,3	0,115	
4	18,199	0,2876	4,871	0,0763	123,3	6,5	4,5317	
5	20,321	0,0163	4,367	0,0035	178,2	8,7	0,346	
6	22,452	0,1902	3,957	0,0331	112,2	6,1	3,49	
7	25,971	0,0203	3,428	0,0026	120,7	7,1	0,2633	
* d-interplanar distances; ** esd-error; *** FWHM- full width at half maximum.								

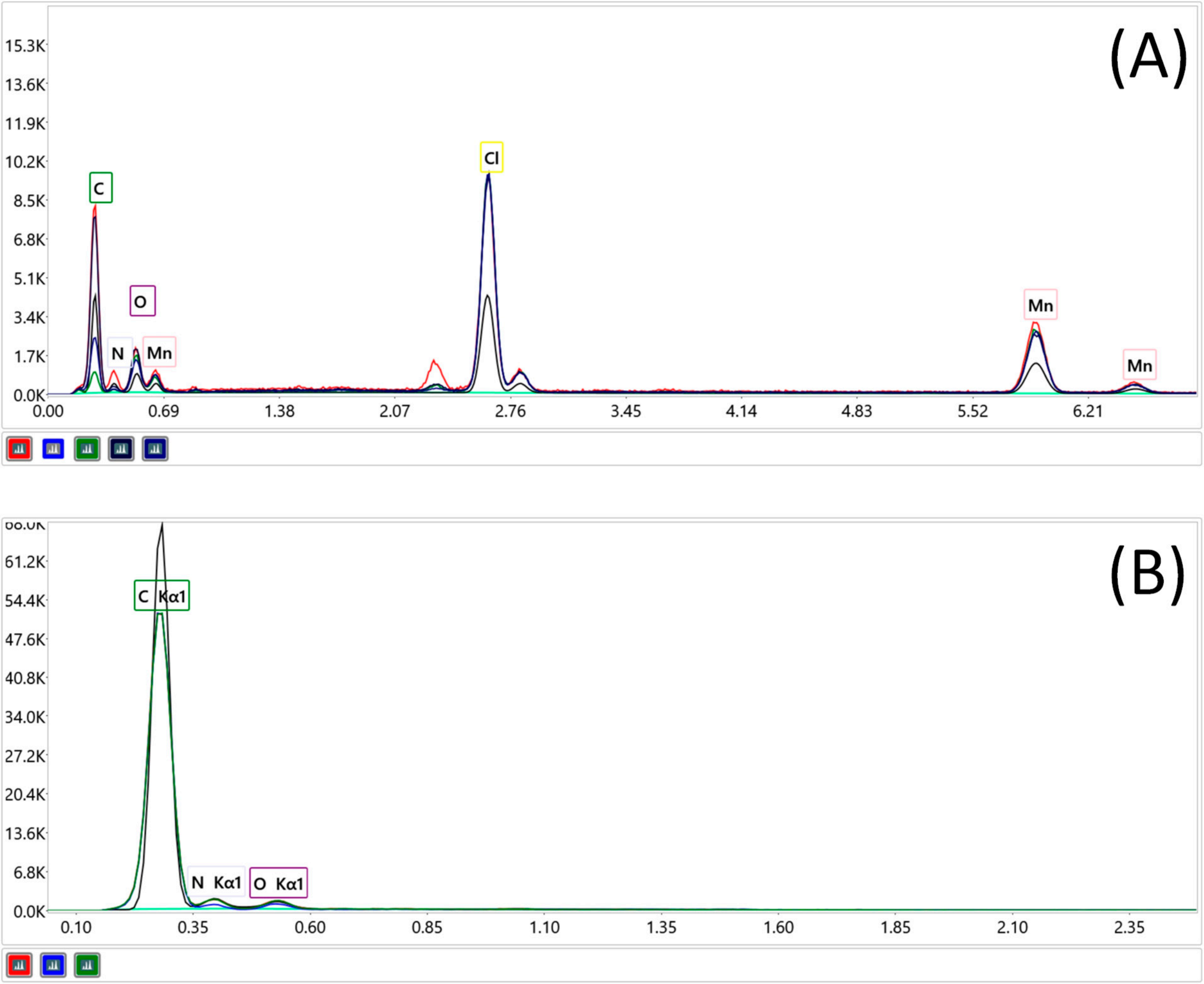

Figure S4. EDX spectra of Mn-CNDs (A) and DPH-CNDs (B) samples.

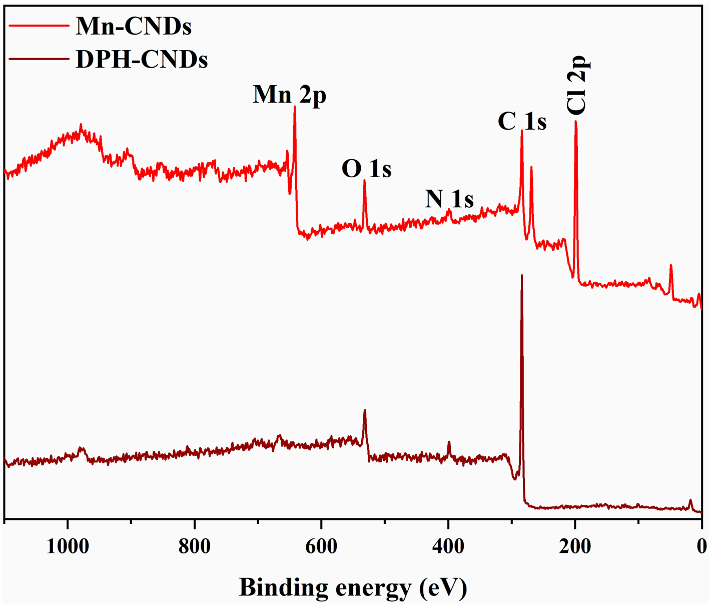

Figure S5. Wide XPS spectra recorded for the prepared DPH-CNDs and Mn-CNDs.

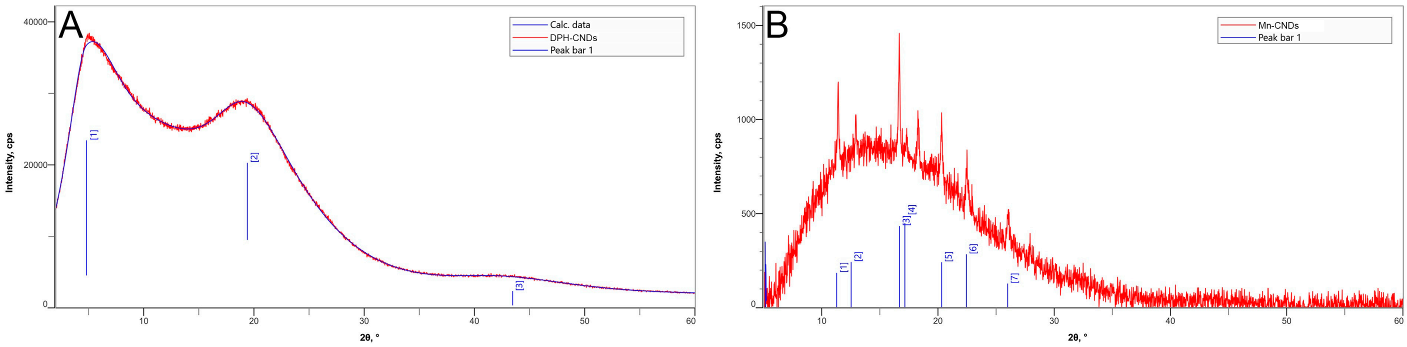

Figure S6. Powder XRD diffractograms of DPH-CNDs with background correction (A), Mn-CNDs without background correction, with an insert showing the expanded diffractogram between 24-300 2θ (B) and Mn-CNDs with background correction (C).

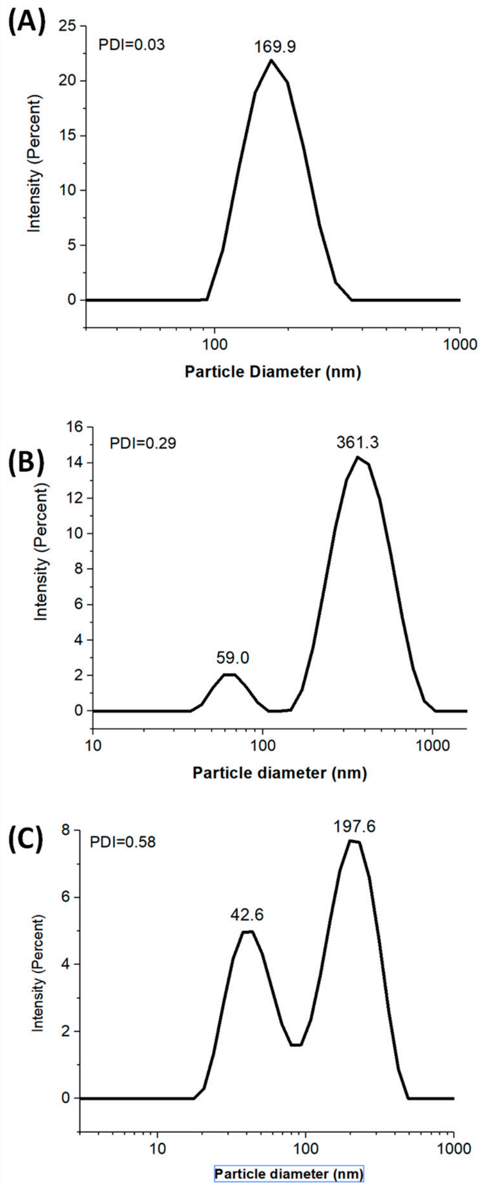

Figure S7. DLS measurements of Mn-CNDs dispersed in ethanol (A), ethanol:water (1:1 v/v) (B), and water (C).

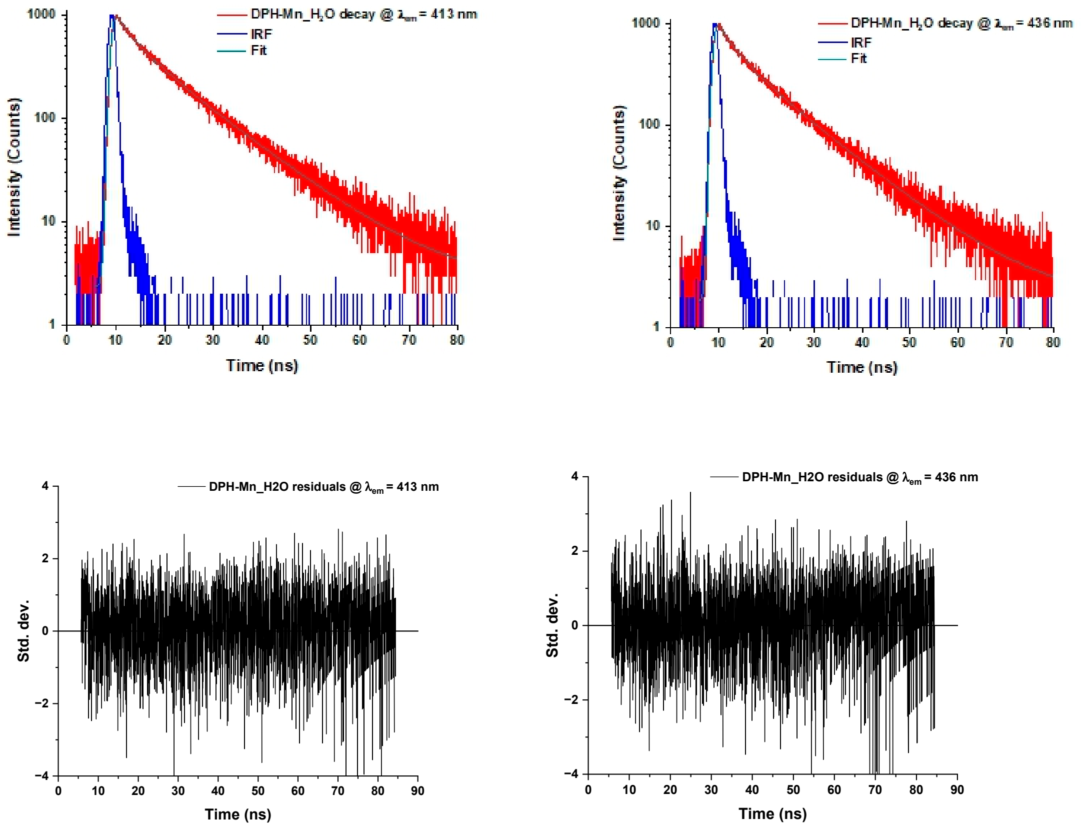

Figure S8. Photoluminescence lifetime decay recorded for the Mn-CNDs at 413 and 436 nm emission peaks.

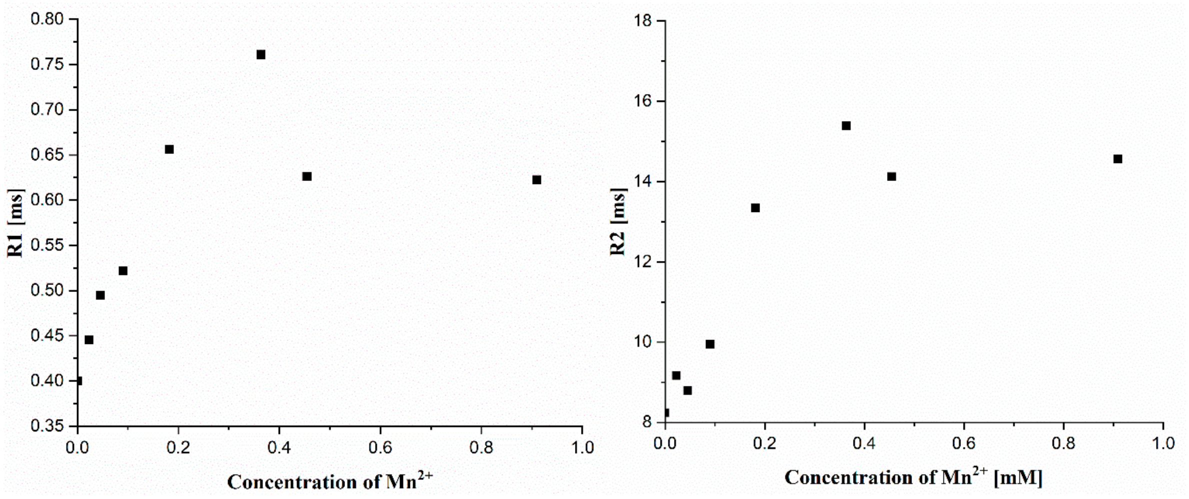

Figure S9. The dependence of R1(1/T1) and R2(1/T2) on the concentration of Mn2+ ions.

At the concentrations of 0.1 and 0.2 mg/mL (0.45 and 0.9 mM Mn), both R1 and R2 decreased and no linear fit can be plotted on the entire range of concentrations.
